# EDTA‐induced pseudothrombocytopenia in hematopoietic stem cell donor

**DOI:** 10.1002/ccr3.7023

**Published:** 2023-04-05

**Authors:** Misato Tsubokura, Minoru Kojima, Saori Nakabayashi, Noriko Takahashi, Sayaka Takeuchi, Yu Aruga, Chiaki Ikeda, Naoki Maezawa, Koji Okazaki, Sanshiro Uchida, Mizuki Watanabe, Jun Aoki, Ayumu Ito, Takashi Tanaka, Yoshihiro Inamoto, Sung‐Won Kim, Hiromichi Matsushita, Takahiro Fukuda

**Affiliations:** ^1^ Department of Laboratory Medicine National Cancer Center Hospital Tokyo Japan; ^2^ Department of Hematopoietic Stem Cell Transplantation National Cancer Center Hospital Tokyo Japan

**Keywords:** EDTA, hematopoietic stem cell transplantation, peripheral blood stem cell harvest, pseudothrombocytopenia

## Abstract

We herein report a case of peripheral blood stem cell transplantation (PBSCT) involving a donor with EDTA‐induced pseudothrombocytopenia (PTCP). The apheresis product was inspected for 24 h and there was no platelet clumping or thrombocytopenia. In the first 14 months after PBSCT, there has been no transfer of PTCP symptoms.

## Abstract 2

In our case, pseudothrombocytopenia (PTCP) symptoms were not transferred from a stem cell donor to the recipient, and the apheresis products harvested with appropriate anticoagulants did not exhibit PTCP. Peripheral blood stem cell transplantation involving PTCP donors may be managed identically to that involving non‐PTCP donors.

## 1 INTRODUCTION

Pseudothrombocytopenia (PTCP) is an in vitro reaction in which a spuriously low platelet count results from blood anticoagulation due to in vitro platelet agglutination, mainly following the use of ethylenediaminetetraacetic acid (EDTA), which chelates divalent cations such as calcium to prevent coagulation. EDTA‐induced PTCP arises from a platelet autoantibody targeting a concealed epitope on the platelet membrane glycoprotein (GP) IIb/IIIa; this epitope becomes exposed by EDTA‐induced dissociation of GP IIb/III.^1^, ^2^ Microscopic examination can identify platelet clumping, and repeat tests using heparin or citrate as an anticoagulant can confirm the diagnosis. This in vitro artifact usually persists over the course of long‐term follow‐up. Three studies thus far have evaluated the prevalence of PTCP in blood and platelet apheresis donors, with frequencies ranging from 0.01% to 0.2%.^3^, ^4^, ^5^ In allogeneic hematopoietic stem cell transplantation (HSCT), autoimmunity and allergy can be transferred from donors to recipients, and this includes autoimmune diseases.^6^, ^7^ B cells do not become fully reconstituted until at least 1–2 years after HSCT, mainly because of graft‐versus‐host disease (GVHD) prophylaxis and treatment using immunosuppressive drugs. However, mature B cells are transferred from donors to recipients. Therefore, it is possible to transfer PTCP during HSCT, but there is minimal information on this phenomenon so far. Herein, we present the clinical course of a patient who underwent peripheral blood stem cell transplantation (PBSCT) involving a donor with EDTA‐induced PTCP.

## 2 CASE PRESENTATION

The patient was a 39‐year‐old Japanese man who was admitted to a local hospital with a chief complaint of swelling of the gingiva and cervical lymph nodes. The patient underwent an excisional biopsy of a cervical lymph node and was diagnosed with extranodal NK/T‐cell lymphoma nasal type (ENKTL). He was initially treated with radiotherapy and dexamethasone, etoposide, ifosfamide, and carboplatin (RT‐DeVIC), and achieved complete response (CR). One and a half years after treatment, he was admitted to a local hospital due to fever. Peripheral blood evaluation revealed pancytopenia, liver dysfunction, and re‐elevation of sIL2R and EBV DNA. Bone marrow biopsy revealed ENKTL relapse. He began the DeVIC regimen and was admitted to our hospital with the plan of allogenic HSCT using peripheral blood from an HLA‐identical and gender‐mismatched sibling donor, who was accepted as a PBSCT donor after a medical examination. Of note, the donor's platelet count was 157 × 10^9^/L (normal range: 158 × 10^9^/L to 348 × 10^9^/L) at her first visit. The day after the donor started filgrastim, her platelet count decreased to 62 × 10^9^/L, and platelet aggregation was observed on a peripheral blood smear. The platelet count was re‐evaluated immediately after blood was drawn and anticoagulated with EDTA or citrate acid (CA), and was found to be 209 × 10^9^/L with EDTA and 202 × 10^9^/L with CA. The thrombocytopenia was considered to represent EDTA‐induced PTCP, in which the platelet count decreases with time after the blood is drawn. After that, until the peripheral blood stem cell harvest (PBSCH), there was no decrease in platelet count when it was measured immediately after blood sampling. PBSCH was performed on the fourth day of treatment with high‐volume granulocyte colony‐stimulating factor (G‐CSF), using combined anticoagulation with heparin and acid‐citrate‐dextrose solution A for the continuous mononuclear cell protocol on the Spectra Optia® (Terumo BCT). A total of 2.3 × 10^6^ CD34+ cells/kg were collected and cryopreserved using dimethyl sulfoxide (DMSO). The apheresis product was inspected for 24 h and there was no platelet clumping or thrombocytopenia with either EDTA or CA evaluation (Table 1). HSCT was performed with a reduced‐intensity conditioning regimen (fludarabine 30 mg/m^2^ and melphalan 140 mg/m^2^, and GVHD prevention with tacrolimus and methotrexate), and after three courses of the DeVIC regimen the patient achieved CR as determined by PET‐CT. There were no hematopoietic stem cell infusion‐related adverse events. Granulocyte engraftment occurred on day 14, and platelets engrafted on day 21. The lymphocyte count recovered steadily, and the CD19+ B‐lymphocyte count recovered to over 300/μL at 6 months after PBSCT. Acute GVHD began on day 19, and developed into grade II and gut stage 1. The patient responded to treatment with hydrocortisone and beclomethasone dipropionate, and achieved CR. The hydrocortisone and beclomethasone dipropionate were stopped on days 54 and 152, respectively. The onset of chronic GVHD was observed on day 187, with lichenoid mucosal changes in the oral cavity and elevated liver enzymes (skin1 and liver1). Treatment with low‐dose tacrolimus is ongoing 14 months after PBSCT because of chronic GVHD. Both sIL2R and EBV‐DNA in the peripheral blood remain within normal limits. A recent PET scan indicated ongoing CR. The platelet count remains normal, with no platelet aggregation in the presence of either EDTA or CA.

**TABLE 1:** Results of longitudinal inspection of the stem cell harvest from a donor with EDTA‐induced pseudothrombocytopenia.

Inspection time point	Anticoagulant	Platelet (×10^9^/L)	Aggregation
Immediate	EDTA	2009	no
	CA	1808	no
2 h later	EDTA	2049	no
	CA	1789	no
3 h later	EDTA	2078	no
	CA	1785	no
4 h later	EDTA	2076	no
	CA	1821	no
5 h later	EDTA	2104	no
	CA	1807	no
6 h later	EDTA	2136	no
	CA	1835	no
18 h later	EDTA	2304	no
	CA	1969	no
24 h later	EDTA	1760	no
	CA	1747	no

Abbreviations: CA: citric acid, EDTA: ethylenediaminetetraacetic acid.

## 3 DISCUSSION

PTCP may begin after HSCT when the B‐cell repertoire develops, but in our case, PTCP was not transferred from the stem cell donor to the recipient. Only one previous case report has described the risk of transmission of PTCP from donor to recipient in the context of HSCT.^8^ In that report, evaluation on day 280 after HSCT revealed no PTCP. A study of plateletpheresis donors found no transfer of PTCP to recipients.^5^ In another study of blood donors with PTCP, no post‐transfusion reactions occurred in recipients, and fresh‐frozen plasma did not cause PTCP.^4^ Traceback was performed in seven recipients of fresh‐frozen plasma from donors with EDTA‐dependent antibodies, and none exhibited a decreased platelet count. Considering these reports, PTCP is probably not transferred from donor to recipient by HSCT or transfusion. In addition, apheresis products harvested with appropriate anticoagulants did not exhibit PTCP despite observation for over 24 h. This is the first report in which apheresis products from a PTCP donor were inspected, and it revealed no platelet clumping or thrombocytopenia. These data suggest that PTCP should not be regarded as an exclusion criterion for stem cell donation.

Despite only a few relevant reports, including our own, there has been no evidence of PTCP transfer from stem cell donors to recipients. Additional more large number data should be accumulated to determine the likelihood of transferring PTCP to patients undergoing HSCT.

## AUTHOR CONTRIBUTIONS

M.T. and M.K. contributed to the conceptualization, investigation, and writing of the original draft. S.N., N.T., S.T., Y.A., C.I., K.O., S.U., M.W., J.A., A.I., T.T., Y.I., and S.W.K. contributed to the investigation and manuscript review and editing. N.M., H.M., and T.F. contributed to manuscript review and editing and supervision.

## FUNDING INFORMATION

This work was supported by a grant from the National Cancer Research and Development Fund (2020‐A‐15).

## CONFLICT OF INTEREST STATEMENT

The authors have no competing interests to declare.

## CONSENT

Written informed consent was obtained from the patient to publish this report in accordance with the journal's patient consent policy.

## ETHICS STATEMENT

This study was approved by the Institutional Review Board of the National Cancer Center. All procedures performed in studies involving human participants complied with the ethical standards of the institutional and/or national research committee and with the 1964 Helsinki Declaration and its later amendments or comparable ethical standards.

## Data Availability

The data that support the findings of this study are available from the corresponding author upon reasonable request.
